# A Lowlands Perspective on Exaggeration and Feigned Symptoms

**DOI:** 10.1192/j.eurpsy.2022.156

**Published:** 2022-09-01

**Authors:** B. Dandachi-Fitzgerald

**Affiliations:** Universiteitssingel, Clinical Psychological Science, Maastricht, Netherlands

**Keywords:** Symptom validity, Performance validity, Feigning, Symptom Exaggeration

## Abstract

Some patients present symptoms in an exaggerated manner [1,2]. This behavior can be assessed with specialized tests: Symptom validity tests (SVTs) to measure overreporting of symptoms, and performance validity tests (PVTs) to measure underperformance on cognitive tests. But what does it mean when patients fail on multiple SVTs and/or PVTs? Does it reflect malingering; i.e. grossly exaggerating or feigning symptoms to gain an external benefit? Could it be seen as a plea for help in some cases? Or could pain, fatigue or cognitive impairment be underlying reasons for the validity test failures? In this presentation some credible and non-credible explanations for failing on validity tests will be discussed. A tentative framework that might aid in conceptualizing poor symptom validity will be presented. References [1] Dandachi-FitzGerald, B., Merckelbach, H., Bošković, I., & Jelicic, M. (2020). Do you know people who feign? Proxy respondents about feigned symptoms. Psychological Injury and Law, 13, 225–234. [2] Merckelbach, H., Dandachi-FitzGerald, B., van Helvoort, D., Jelicic, M., & Otgaar, H. (2019). When patients overreport symptoms: More than just malingering. Current Directions in Psychological Science, 28, 321–326.

**Disclosure:**

No significant relationships.

